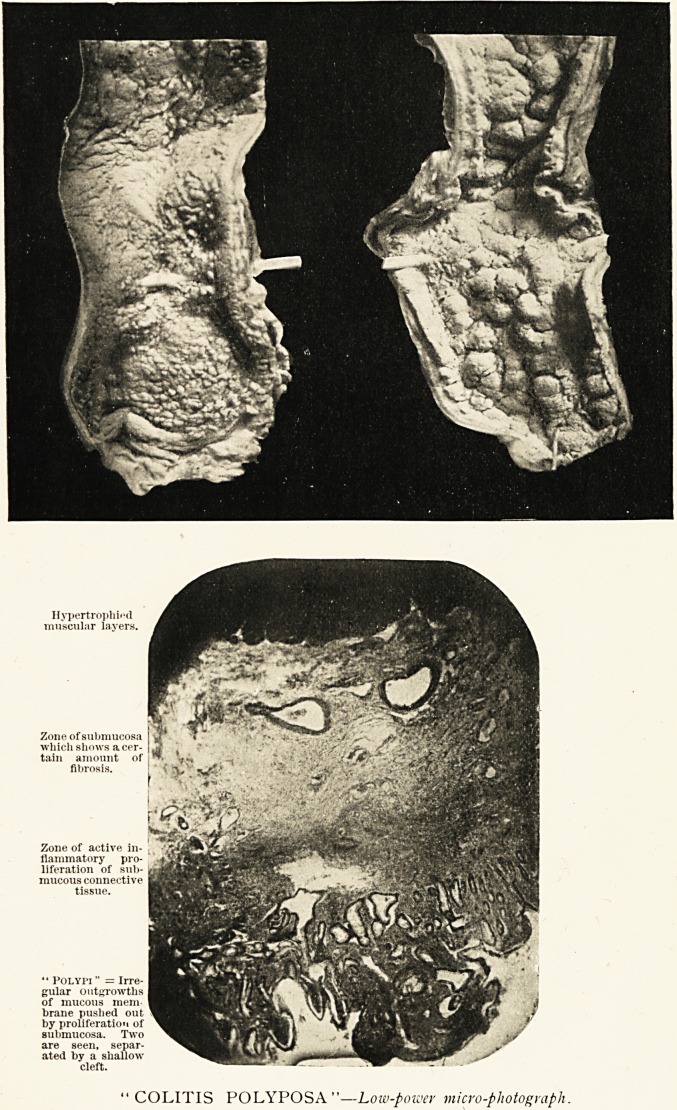# Colitis Polyposa

**Published:** 1906-03

**Authors:** Carey Coombs

**Affiliations:** Curator of the Museum, Bristol General Hospital; Physician to Out-patients, Royal Hospital for Sick Children and Women, Bristol


### COLITIS POLYPOSA.

BY

CAREY COOMBS, M.D., B.S. Lond.,

*Curator of the Museum, Bristol General Hospital; Physician to Out-patients, Royal Hospital for Sick Children and Women, Bristol.*

---

THE subject of this paper is a specimen in the museum of the Bristol General Hospital, which seems worthy of attention because it is an example of a condition hitherto only described once, I believe, by Dr. Pope, of Leicester.[1]

It was taken from a patient admitted to the General Hospital in October, 1900, under Dr. Harrison, who has kindly allowed me to make use of the clinical notes. I am also much indebted to Dr. Michell Clarke for further details and for permission to use his *post-mortem* notes. The patient was a servant girl, 29 years old, who in August, 1900, began to have a frequent desire to evacuate the bowels. Her motions became "matter-like" and loose, and often contained blood; she had indeed passed blood before this, but up till August had given little heed to it. There was some abdominal pain of an aching character, never very intense. She had never been abroad, and gave no history of previous illnesses or of anything which could in any way account for the onset of her present troubles. When admitted she was anæmic, feeble and wasted. There had been, on an average, four or five motions daily, and though at first it seemed as if under treatment she were improving, this was only temporary, and the diarrhœa continued without intermission till her death in January, 1901. Much mucus was passed, and often there was blood in the stools. The temperature fluctuated between 98° and 101° throughout her stay in hospital. Dr. Clarke tells me that during life the colon could be felt through the abdominal wall. Clinically, therefore, the case in most respects imitated the course of chronic dysentery.

[1] Pope, *Brit. M. J.*, 1904, ii. 180.

At the autopsy the body was wasted and remarkably free of fat. The small bowel was thin and atrophic, and the morbid process was limited to the large intestine, which was thick and firm, with a narrowed lumen. There was neither perforation, peritonitis, nor enlargement of lymphatic glands. The specimen about to be described consists of the cæcum, the ascending and part of the transverse-colon. When fresh it was pale red in colour. The wall of the bowel is seen to be thick; this thickening is mainly due to increase in depth of the structures internal to the muscular layers, though these also have undergone hypertrophy by reason of the excessive frequency of peristalsis. It is the inner aspect of the bowel that is especially abnormal. At the cæcal end of the specimen the usual appearance of the mucous membrane is entirely wanting, and instead there are to be seen a great number of small polypi thickly set all over the internal surface of the bowel, with practically no open space intervening between them. They vary in size from that of an ordinary pin's head to that of small shot, the larger ones being distinctly stalked. The further from the cæcum, speaking generally, the smaller are the polypi; they are, however, gathered together into round masses, the larger of which have an area equal to that of a threepenny-piece. These masses are separated from each other by linear fissures, which seem to be merely clefts between the protuberances of polypoid mucous membrane, though some of them may be partly the result of ulceration. Nowhere is there ulceration deep enough to excavate the submucous coat to any extent, but there are a few superficial ulcers eroding the summits of the polypi, especially at the colon end of the specimen.

Microscopically the striking feature of the section is the increase in thickness of the submucous coat, which is everywhere about half as thick again as in the normal colon. It is this increase in the submucosa which has resulted in the polypoid state of the mucosa; the overgrowth of the connective tissue beneath the mucous membrane has pushed it out into a series of closely-set, irregular papillary processes. The clefts between the polypi represent those parts where the mucous membrane has not been pushed out, and do not seem to be due to cicatricial contraction of the submucosa. The submucosa is seen to be divided into two layers: the outer of these is of normal consistence, except for an unimportant degree of round-cell infiltration in the neighbourhood of blood-vessels ; this is to be seen also in the muscular and even in the subserous layers. The part of this outer zone of submucosa which immediately borders on the inner zone is rather denser in texture than normal, owing to the presence of inflammatory fibrous and fibroblastic tissue. The inner and immediately subepithelial zone shows a much greater degree of morbid change, and it is evidently to this layer that the greater part of the submucous thickening must be ascribed. There is an intense round-cell infiltration of this region, most intense about the neighbourhood of the muscularis mucosæ (which is so disorganised as to be scarcely visible) and slightly less so in the finger-like outgrowths which constitute the polypi. The round cells are for the most part mononuclear, and probably arise from the proliferation of the fixed cells of the tissue, except in one or two places immediately beneath the epithelium, where the inflammatory tissue has broken down: at these spots the cells are many of them polymorphonuclears, so possibly these areas represent the result of secondary infection by the various inhabitants of the large bowel. A few small vessels, chiefly veins, are thrombosed, and there is proliferation of the endothelium in some of the capillaries and smaller arterioles.

With regard to the epithelium, it is considerably distorted by the swelling and overgrowth of connective tissue beneath it; it is pushed out into the lumen of the bowel to form polyps which are riddled with crypts, the result of the irregular outgrowth of the submucosa; these crypts, on section, have the appearance of spaces lined by columnar and cuboidal epithelium, some of the larger of which probably represent cystic dilatation of the crypts due to obstruction by cicatrising submucous tissue. The total epithelial surface is, of course, greatly increased, but only as a result of the overgrowth of submucosa. The epithelial cells themselves are for the most part well formed; in some of the cystic spaces they are flattened, in some spots they have undergone mucoid degeneration, and in some of the recesses between the polypi the surface is denuded of cells.

The process, then, to sum up, is a chronic inflammatory hyperplasia of the submucous layer of the colon, with consequent deformity of the epithelial surface. Dr. Pope's patient was a girl of 21, who had abdominal pain, constipation alternating with diarrhœa, and dysenteric stools for six months before death. *Post-mortem*, the whole colon was found covered with small polypi, and the microscope showed that the predominant change was an inflammatory hyperplasia of the submucosa. The specimen has since been included in the museum of the Royal College of Surgeons, where Mr. Shattock, the curator, tells me it is unique. In all their essential features, the case I have described and Dr. Pope's case are similar; both were young women, both succumbed after about six months of what appeared to be chronic dysentery, that is to say, diarrhœa (in one case alternating with constipation) with passage of blood and much mucus. In both cases exhaustion steadily progressed till it reached a fatal degree. *Post-mortem*, there was in both a general polyposis of the mucous membrane of the colon due to chronic inflammatory overgrowth of the submucous coat, while ulceration was absent or unimportant in degree. In both the rectal end of the colon had suffered less than the cæcal end.

Like Dr. Pope, I have been unable to find any record of a similar state. It is noteworthy that several of the newer "jerry-built" text-books allude vaguely to polyposis of the mucous membrane of the colon as a result of chronic catarrh, while more reliable works are silent on this subject. In Wilks and Moxon's book,[1] it is true, the term "Colitis polyposa" is used, but here and also in St. Bartholomew's Hospital Museum[2] it is applied to quite a different condition, namely to an advanced stage of ulcerative colitis in which such parts of the mucous membrane as have been spared by the destructive

1 Wilks and Moxon, *Pathological Anatomy*, 1889, p. 434.
2 Specimens 1987a, b, and c. *Vide St. Barth. Hosp. Rep.*, 1889, xxv. 306.

process are polypoid in form. In this condition, doubtless, the surviving masses of mucous membrane owe part of their bulk to chronic inflammation; the picture, however, of such a colon is that of ulceration with accidental polyposis, not of polyposis with unimportant ulceration. In Ziegler's book [1] is an excellent figure representing this; while a picture in Hemmeter's work, labelled "Colitis polyposa," seems to portray the same thing.[2] It is unfortunate that the same name should have been given to two quite distinct morbid processes; the condition exemplified by my specimen seems, however, to be more deserving of an individual name than a particular phase of ulcerative colitis; and the term "polypus" applies more aptly to that which is heaped up by a productive inflammation than to that which is the survivor of a destructive process.

There appears to be no very close alliance between this condition and the multiple adenomatous polypi sometimes found in the colon and also in the small bowel; the adenomatous polyps are larger and less crowded, while the specimen described here is undoubtedly representative of a chronic inflammatory process, though it is conceivable that the formation of adenomata might be preceded by an inflammatory stage. More closely similar are those results of chronic inflammation of the gastric wall known respectively as "état mamelloné" and "cirrhosis of the stomach." In the former there is a general polyposis of the mucosa, while in the latter, though there is glandular atrophy, there is great hyperplasia with fibrosis of the submucous coat. Bristowe says [3] that a similar cirrhosis may occur in the intestine, but makes no further allusion to it. Ruffini, of Bologna,[4] published a case of somewhat similar nature under the name of "diffuse hyperplastic colitis"; the patient was a woman of 38, who for a year or two before death (which occurred shortly after childbirth) had suffered from bouts of diarrhœa with abdominal pains, wasting, anæmia, and œdema of the lower limbs. *Post-mortem*, the wall of the colon

[1] Ziegler, *Special Pathological Anatomy* (trans. by MacAlister and Cattell), Sect. ix.–xv., 1897, p. 660.

[2] Hemmeter, *Diseases of the Intestines*, vol. i., 1901, p. 713.

[3] Bristowe, *Text-book of Medicine*, 7th ed., 1890, p. 703.

[4] Ruffini, *Bull. d. Sc. med. di Bologna*, 1892, iii. 147.

was very thick owing to cellular infiltration of the submucous coat, especially marked just beneath the epithelium; the vessels of the submucous coat were thrombosed, which Ruffini supposed to be the primary stage in the process. Dr. Pope thought his patient's condition was in some way due to a purulent vulvo-vaginitis from which she suffered, but in the case recorded here there was absolutely nothing to which the inception of the disease could be ascribed. Perhaps it merely represents the final phase of what we are accustomed to call a simple catarrh of the bowel, though as this is common and colitis polyposa would appear to be an unusual matter, it is not likely that simple catarrh alone was responsible. It may be, on the other hand, the achievement of some specific irritant, perhaps a simple body such as mercury or arsenic, or even some parasite which only occasionally makes man its host. Whatever the cause, the condition itself is worthy enough of note, especially when in the only two recorded instances there is so close a clinical as well as pathological similarity.

In conclusion, I must express my thanks to Mr. F. F. Leighton for his excellent microscopical sections, and to Mr. G. R. Rew for a low-power photo-micrograph.

*Description of Plate.*

The left-hand portion is the cæcum and beginning of the ascending colon, in which the polypi are seen to be larger and arranged singly. The right-hand portion is a part of the transverse colon; here may be seen rounded masses of smaller polypi, separated by linear fissures. The cut surface in the left upper part of the right-hand specimen shows the thickening of the intestinal wall, and especially of the submucous layer.

Hypertrophied muscular layers.

Zone of submucosa which shows a certain amount of fibrosis.

Zone of active inflammatory proliferation of submucous connective tissue.

" POLYPI " = Irregular outgrowths of mucous membrane pushed out by proliferation of submucosa. Two are seen, separated by a shallow cleft.

" COLITIS POLYPOSA "—*Low-power micro-photograph.*

## Figures and Tables

**Figure f1:**